# Sphenoid sinus osteitis after endoscopic transsphenoidal surgery: a bone-centered postoperative entity

**DOI:** 10.1007/s00701-026-06880-w

**Published:** 2026-04-27

**Authors:** Burak Çabuk, Atılay Yaylacı, Fatih Shatri, Sertaç Ayyıldız, Eren Yılmaz, Anıl Ergen, Melih Çaklılı, İhsan Anık, Savaş Ceylan

**Affiliations:** 1 Department of Neurosurgery, Kocaeli University, Kocaeli, Türkiye 41001; 2 Department of Otorhinolaryngology, Kocaeli University, Kocaeli, 41001 Türkiye; 3 Department of Neurosurgery, Istinye University, Istanbul, 34480 Turkey; 4 Department of Neurosurgery, Bahcesehir University School of Medicine, Istanbul, 34353 Turkey

**Keywords:** Osteitis, Endoscopic, Skull base, Sfenoid sinus, Sinusitis, Pituitary

## Abstract

**Purpose:**

Despite advances in endoscopic skull base surgery, postoperative sinonasal inflammation remains poorly characterized. In particular, sphenoid sinus osteitis has received little attention, as complications after endoscopic transsphenoidal surgery (ETS) have largely been assessed in terms of mucosal pathology. This study aims to determine the incidence of sphenoid sinus osteitis after ETS, describe its clinical and radiological features, and identify associated surgical and demographic risk factors.

**Methods:**

We retrospectively reviewed patients undergoing primary ETS for sellar or parasellar tumors between September 2021 and September 2024. Osteitis was assessed on high-resolution CT using the Lee and Kennedy classification. Clinical symptoms, particularly cacosmia, and surgical approach and reconstruction techniques were analyzed as potential risk factors.

**Results:**

Among 1,443 patients, sphenoid sinus osteitis was identified in 12 cases (0.83%). All cases occurred in patients who underwent sellar reconstruction, with no osteitis observed in those without reconstruction (*p* < 0.001). Regarding reconstruction techniques, the nasoseptal flap showed a borderline statistical significance (*p* = 0,045). Radiologically, osteitis was Grade 1 in 50%, Grade 2 in 25%, and Grade 3 in 25% of cases. Cacosmia was the presenting symptom in 75% of affected patients (*p* < 0.001). Surgical revision was required in 11 patients (91.7%), while medical treatment alone was sufficient in one case. At the 12-month follow-up, all of the patients were symptom-free; one patient required a second revision for recurrent sphenoid sinusitis at sixth months follow up.

**Conclusion:**

Sphenoid sinus osteitis represents a rare but clinically relevant bone-centered complication following ETS, distinct from conventional rhinonasal morbidity. Its exclusive association with sellar reconstruction highlights the potential role of reconstruction-related bone contact and localized inflammatory responses in its pathogenesis. Recognizing sphenoid sinus osteitis as a distinct postoperative entity may improve diagnostic accuracy and guide more targeted management of postoperative rhinonasal symptoms. Surgical revision served as the primary intervention for the patient cohort, yielding favorable outcomes characterized by complete symptomatic resolution at the 12-month follow-up.

## Introduction

Endoscopic transsphenoidal surgery has become the standard surgical approach for the treatment of pituitary adenomas and other sellar and parasellar pathologies [4, 17, 34]. Advances in endoscopic techniques and skull base reconstruction have significantly improved surgical outcomes; however, postoperative sinonasal and skull base–related complications continue to affect patient comfort, quality of life, and long-term surgical success [7].

In the existing literature, postoperative complications following endoscopic transsphenoidal surgery have predominantly been evaluated under the headings of sinonasal infections, mucosal inflammation, crusting, and sinusitis, with reported rates reaching up to 3–4%. [7, 22, 30] While these studies provide valuable information regarding mucosal morbidity, the potential long-term effects of these inflammatory processes on the underlying sphenoid sinus bone have received little attention. Nevertheless, it is well established that chronic inflammatory conditions may involve not only the mucosa but also the adjacent bone tissue [1, 11, 33].

Osteitis represents an inflammatory reaction of bone and has been described using terms such as hyperostosis, bony involvement, and neo-osteogenesis [1, 19, 20, 32]. In the context of chronic rhinosinusitis, persistent mucosal inflammation has been shown to induce structural changes in the adjacent bone, including sclerosis and thickening, as demonstrated by both radiological and histopathological studies [1, 28]. In neurosurgical practice, osteitis is commonly discussed in relation to cranioplasty and craniotomy flap infections, where the use of synthetic materials and compromised bone vascularity play a critical role [14, 25, 35].

During endoscopic transsphenoidal and extended endonasal skull base procedures, various reconstruction techniques—including collagen-based dural substitutes, autologous fascia lata grafts, and vascularized nasoseptal flaps—are routinely employed to prevent cerebrospinal fluid leakage [13, 17, 24, 30, 34]. Although these techniques are essential for surgical success, their direct contact with the sphenoid sinus bony walls may trigger inflammatory bone responses. Despite this potential mechanism, sphenoid sinus osteitis following endoscopic transsphenoidal surgery has not been systematically investigated as a distinct, bone-centered complication, as existing studies have largely focused on mucosal pathology [7, 16, 22, 30].

Therefore, the aim of this study is to determine the incidence of sphenoid sinus osteitis in a large cohort of patients undergoing endoscopic transsphenoidal surgery, to characterize its clinical and radiological features, and to analyze potential demographic and surgical risk factors associated with its development.

## Materials and methods

### Study design and patient selection

This study was designed as a retrospective cohort study and conducted in accordance with the ethical standards of the institutional research committee and with the principles of the Declaration of Helsinki. Ethical approval was obtained from the Kocaeli University Faculty of Medicine Clinical Research Ethics Committee (GOKAEK-2025/22/36). Prior to surgery, written surgical informed consent was obtained from all patients, which included permission for the use of anonymized hospital records for scientific research purposes. Due to the retrospective nature of the study and the use of anonymized data, the requirement for additional study-specific informed consent was waived by the ethics committee. Medical records of patients who underwent endoscopic transsphenoidal surgery for sellar or parasellar tumors between September 2021 and September 2024 were retrospectively reviewed.

During the study period, a total of 1,908 patients underwent endoscopic transsphenoidal surgery. Inclusion criteria consisted of patients who had undergone primary endoscopic transsphenoidal surgery for a sellar or parasellar tumor. Patients were excluded if they had a history of previous transsphenoidal or paranasal sinus surgery, preoperative imaging evidence of sphenoid sinus pathology (such as sinusitis or mucocele), or insufficient postoperative follow-up data.

### Definition of postoperative osteitis

The primary endpoint of the study was the presence of postoperative sphenoid sinus osteitis. The diagnosis of osteitis was established based on a combination of radiological findings and clinical symptoms, as detailed below.

### Radiological evaluation

All patients had preoperative sellar magnetic resonance imaging (MRI) and paranasal sinus computed tomography (CT) scans available for evaluation. According to the institutional postoperative follow-up protocol, cranial MRI was routinely performed at 1, 3, and 6 months postoperatively, at 1 year, and annually thereafter.

Patients who demonstrated suspicious sphenoid sinus mucosal thickening or contrast enhancement on follow-up MRI, as well as those with clinical suspicion of osteitis, underwent high-resolution paranasal sinus CT to allow detailed assessment of bony structures. Postoperative CT scans of patients with suspected osteitis were evaluated according to the radiological osteitis classification proposed by Lee and Kennedy (2006) [19].

In this system, osteitis is graded for each sinus (excluding the frontal sinus) based on the degree of bony thickening and sclerosis as follows: Grade 1 (mild): bone thickness up to 3 mm, Grade 2 (moderate): bone thickness of 4–5 mm, Grade 3 (severe): bone thickness greater than 5 mm.

High-resolution CT images were independently evaluated by two observers. In cases of disagreement, a consensus was reached to determine the final grade. When necessary, MRI findings were correlated with CT images to improve diagnostic accuracy.

### Clinical evaluation

All patients were systematically evaluated during postoperative follow-up visits for symptoms potentially associated with osteitis. Particular emphasis was placed on cacosmia (persistent foul odor perception), a symptom known to significantly impair postoperative quality of life following endoscopic transsphenoidal surgery. At each follow-up visit, patients were actively questioned regarding the presence, onset, and severity of cacosmia, as well as its impact on daily activities.

Additional symptoms, including persistent nasal obstruction, purulent postnasal discharge, and atypical headache, were also recorded. Patients presenting with these symptoms underwent further evaluation with paranasal sinus CT. Clinical findings were correlated with radiological results to support the diagnosis of postoperative osteitis.

### Surgical approach

Demographic data (age and sex), comorbidities, and were recorded for all patients. Surgical variables considered potential risk factors for osteitis development were also documented. Based on the anatomical extent of surgical dissection, surgical approaches were categorized into two groups. Middle turbinate resection was not performed in any patient.

Standard endoscopic endonasal approach, defined as procedures in which the surgical corridor remained within the boundaries of the sella turcica and was used for conventional pituitary lesions. Extended endoscopic endonasal approach, defined as procedures in which the surgical corridor extended beyond the sella turcica to adjacent regions such as the planum sphenoidale, olfactory groove, clivus, or cavernous sinus. These approaches required wider bone and mucosal resection.

### Sellar reconstruction

Sellar reconstruction techniques were classified according to the size of the defect and the risk of cerebrospinal fluid (CSF) leakage. Simple Closure applied in cases with low-risk or suspected low-flow CSF leakage, consisting of placement of a collagen-based dural substitute followed by fibrin sealant application. Multilayer Autologous Grafting used in cases with evident intraoperative CSF leakage, involving multilayer reconstruction with non-vascularized autologous tissues such as fascia lata and/or abdominal fat. Vascularized Flap Reconstruction, Preferred for large skull base defects following extended approaches and in cases of high-flow CSF leakage, using a vascularized nasoseptal flap (NSF).

### Treatment protocol and follow-up for patients with osteitis

All patients diagnosed with postoperative sphenoid sinus osteitis received an initial medical treatment consisting of two courses of oral clarithromycin (each course lasting 10 days) combined with saline nasal irrigation three times daily. Patients who showed clinical and radiological improvement with medical therapy alone continued conservative management. Those who remained symptomatic despite two courses of antibiotic therapy were referred for surgical revision. Following surgical revision, patients were instructed to resume saline nasal irrigation two weeks postoperatively. All patients were followed up with clinical and radiological evaluations at 3, 6, and 12 months after the initial surgery. Endoscopic evaluation was not performed in a standardized manner for all patients but was selectively utilized in cases with persistent or unexplained sinonasal symptoms as part of routine clinical practice.

### Statistical analysis

Statistical analysis was performed using the IBM SPSS Statistics for Windows, Version 26.0 (IBM Corp., Armonk, NY, USA). Descriptive statistics were expressed as mean ± standard deviation (SD) for continuous variables and as frequencies (*n*) and percentages (%) for categorical variables.

The normality of the distribution for continuous variables was evaluated. Comparisons of continuous variables (e.g., age) between the osteitis and non-osteitis groups were performed using the **Student's t-test**. Categorical variables (e.g., sex, surgical approach, reconstruction type) were compared using the **Pearson Chi-square test**. When the expected frequency in any cell of the contingency table was less than 5, **Fisher's exact test** was used. A p-value of less than 0.05 was considered statistically significant.

## Results

Between September 2021 and September 2024, a total of 1,443 patients who underwent endoscopic transsphenoidal surgery and met the study inclusion criteria were retrospectively analyzed. Of these patients, 54.9% were female (*n* = 792) and 45.1% were male (*n* = 651). The mean age of the entire cohort was 44.6 years (range: 18–80). (Table 1) When comparing the osteitis and non-osteitis groups, there were no statistically significant differences regarding mean age (41.92 ± 9.10 years vs. 44.65 ± 13.11 years, *p* = 0.471) or sex distribution (*p* = 0.244).

Regarding surgical approaches, the majority of patients underwent a standard transsphenoidal approach (*n* = 1,341, 93.0%), while an extended approach was performed in a smaller proportion of cases (*n* = 102, 7.0%). Postoperative sphenoid sinus osteitis was identified in 12 patients, corresponding to an overall incidence of 0.83%. The incidence of osteitis was 0.75% (10/1,341) in the standard approach group and 1.96% (2/102) in the extended approach group; however, this difference was not statistically significant (*p* = 0.206).

When reconstruction techniques were evaluated, osteitis was observed exclusively in patients who underwent sellar reconstruction. No cases of osteitis were detected among the 747 patients who did not receive reconstruction (0.0%). Among the specific reconstruction subgroups, osteitis occurred in 5 of 255 patients (1.96%) reconstructed with a nasoseptal flap, 3 of 133 patients (2.26%) receiving fascia lata grafts, and 4 of 308 patients (1.3%) treated with Duragen/Tisseel. Statistical analysis revealed that the use of a nasoseptal flap was associated with a statistically significant difference (*p* = 0.045), whereas the fascia lata (*p* = 0.091) and Duragen/Tisseel (*p* = 0.297) subgroups did not reach statistical significance. (Table 2)

Clinically, cacosmia was present in 9 of the 12 patients with osteitis (75.0%), which was significantly higher than the 1.5% incidence (21/1,410) observed in the non-osteitis group (*p* < 0.001). Among patients reporting cacosmia, culture results did not show a statistically significant difference between negative and positive growth subgroups (*p* = 0.572).

**Fig. 1:**
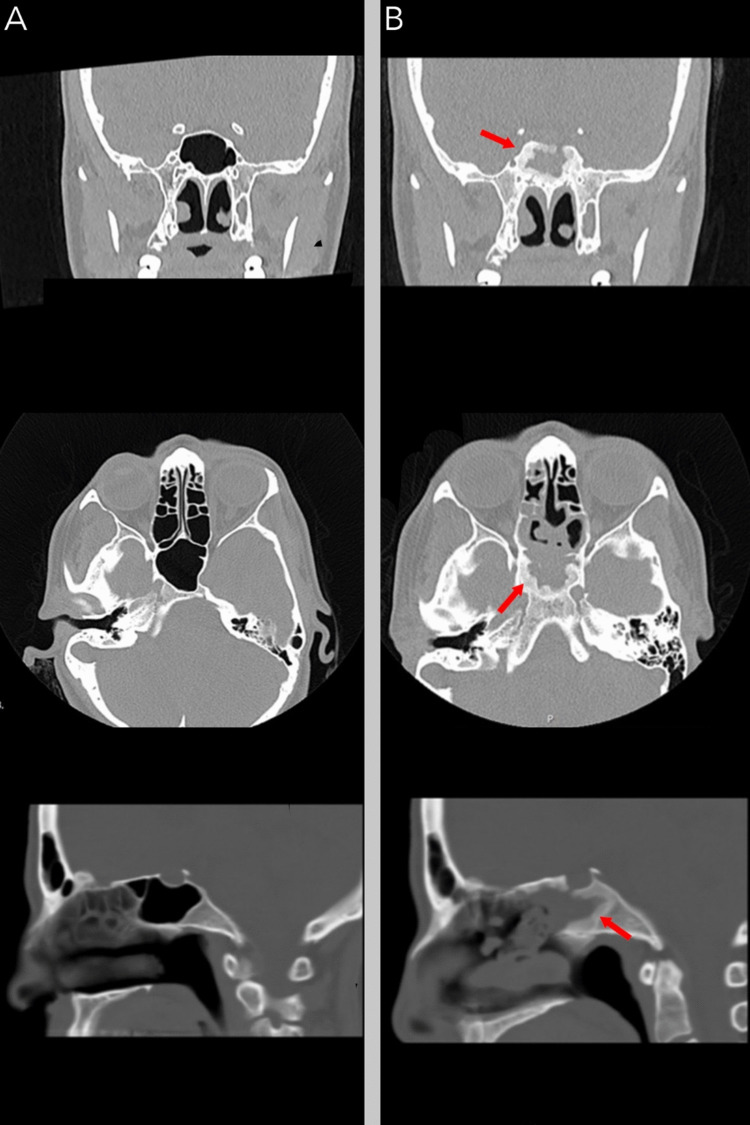
Preoperative (**A**) and postoperative (**B**) computed tomography (CT) images in axial, coronal, and sagittal planes of Patient 12, showing marked bony thickening and sclerosis consistent with Lee–Kennedy Grade 3 sphenoid sinus osteitis. Red arrow: Bony thickening

Radiological evaluation of the 12 osteitis cases according to the Lee and Kennedy classification revealed Grade 1 osteitis in 6 patients (50%), Grade 2 in 3 patients (25%), and Grade 3 in 3 patients (25%). In Grade 2 and higher cases, computed tomography demonstrated cortical irregularity and sclerotic thickening of the sphenoid sinus floor, with associated mucosal contrast enhancement in some patients. In Grade 3 cases, diffuse mucosal thickening accompanying extensive bony involvement was observed. CT findings were dominated by cortical thickening and sclerosis of the sphenoid sinus walls (Fig. 1). No bone marrow edema was detected on magnetic resonance imaging (Table 3).

**Table 1:** Demographic characteristics and surgical distribution

Variable	Value
Total patients	1,443
Female, *n* (%)	792 (54.9)
Male, *n* (%)	651 (45.1)
Age, mean (min–max)	44.6 years (18–80)
Standard approach, *n* (%)	1,341 (92.9)
Extended approach, *n* (%)	102 (7.1)
Total osteitis, *n* (%)	12 (0.83)

**Table 2:** Incidence of osteitis and surgical/reconstruction factors

Variable	Category	No Osteitis (*n* = 1431)	Osteitis (*n* = 12)	*P*-value
Age (Years)	Mean ± SD	44.65 ± 13.11	41.92 ± 9.10	0.471
Sex	Male	648 (45.3%)	3 (25.0%)	0.244*
	Female	783 (54.7%)	9 (75.0%)	
Surgical Approach	Standard	1331 (93.0%)	10 (83.3%)	0.206*
	Extended	100 (7.0%)	2 (16.7%)	
Reconstruction Type	No Reconstruction	747 (52.2%)	0 (0.0%)	-
	Nasoseptal Flap	250 (17.5%)	5 (41.7%)	**0.045**
	Fascia lata	130 (9.1%)	3 (25.0%)	0.091
	Duragen/Tisseel	304 (21.2%)	4 (33.3%)	0.297
Cacosmia	No	1410 (98.5%)	3 (25.0%)	< 0.001*
	Yes	21 (1.5%)	9 (75.0%)	
Culture Result in Cacosmia Patients *(Subset n* = *33)*	Negative	13 (61.9%)	8 (66.7%)	0.572*
	Positive	8 (38.1%)	4 (33.3%)	
Treatment *(Osteitis Group Only)*	Surgery	-	11 (91.7%)	-
	Medical	-	1 (8.3%)	

*Fisher's Exact Test was used. *SD* Standard Deviation

**Table 3:** Clinical, radiological, and treatment characteristics of osteitis-positive patients

Age	Sex	Approach	Reconstruction	Lee grade	Cacosmia	Culture result	PET/CT	Treatment	Outcome at 12 mo
40	F	Standard	NSF	1	No	Candida albicans	No	Surgery	Symptom-free
25	F	Extended	NSF	1	Yes	P. aeruginosa and S. aureus	No	Surgery	Symptom-free
41	F	Standard	NSF	1	Yes	S. marcescens, S. aureus	Right nasal FDG uptake	Surgery	Symptom-free
60	F	Extended	NSF	1	Yes	Unknown	Right nasal FDG uptake	Surgery	Symptom-free
47	M	Standard	*Dur*	1	Yes	Unknown	No	Surgery	Symptom-free
37	M	Standard	*Dur*	3	Yes	S. aureus, K. pneumoniae	No	Surgery	Symptom-free
43	M	Standard	*FL*	3	Yes	Unknown	No	Surgery	Symptom-free
40	F	Standard	*FL*	2	Yes	Unknown	No	Surgery	Symptom-free
39	F	Standard	*FL*	2	No	Unknown	No	Medical	Symptom-free
33	F	Standard	*Dur*	2	No	Unknown	No	Surgery	Symptom-free
54	F	Standard	NSF	1	Yes	No growth	Right nasal FDG uptake	Surgery	Cacosmia resolved after 2nd revision; mild PND
44	F	Standard	*Dur*	3	Yes	Unknown	No	Surgery	Symptom-free

*NSF* Nasoseptal flap, *Dur* Duragen/Tisseel, *FL* Fascia lata, *PND* postnasal drip

Positron emission tomography/computed tomography (PET/CT) was not routinely performed in the study population and was reserved for selected cases with diagnostic uncertainty or for systemic evaluation. PET/CT was used in 2 patients to support the diagnosis and in 1 patient for metastatic screening. In these cases, increased fluorodeoxyglucose (FDG) uptake in the sphenoid region was consistent with the clinical and radiological findings of osteitis.

All 12 patients diagnosed with osteitis initially received two courses of oral clarithromycin (each lasting 10 days) combined with saline nasal irrigation three times daily. In one patient (8.3%), symptoms resolved after two courses of antibiotic therapy, and no surgical intervention was required. The remaining 11 patients (91.7%) underwent surgical revision due to persistent symptoms despite medical therapy. All patients were instructed to resume saline nasal irrigation two weeks after surgical revision. Microbiological cultures were obtained in all 12 patients, with positive growth identified in 4 cases (33.3%). The most frequently isolated microorganism was *Staphylococcus aureus* (*n* = 3). Other isolated organisms included *Klebsiella pneumoniae* (*n* = 1), *Candida albicans* (*n* = 1), *Serratia marcescens* (*n* = 1), and *Pseudomonas aeruginosa* (*n* = 1). Despite negative culture results in the remaining cases, clinical and radiological findings of osteitis were present.

At the 6-month follow-up after the initial surgery, 10 of the 11 surgically treated patients showed complete resolution of symptoms. However, one patient (Patient 11, nasoseptal flap reconstruction, Grade 1 osteitis) presented with persistent cacosmia secondary to recurrent sphenoid sinusitis and underwent a second surgical revision consisting of sphenoid sinus irrigation and debridement. At the 12-month follow-up, all patients were completely symptom-free and no longer required saline nasal irrigation or antibiotic therapy. Patient 11 reported mild intermittent postnasal drip and a sensation of nasopharyngeal fullness; however, the other symptoms had resolved.

## Discussion

The results of the study suggest that sphenoid sinus osteitis represents a rare but distinct postoperative entity following endoscopic transsphenoidal surgery. The exclusive occurrence of osteitis in patients who underwent sellar reconstruction, its association with specific reconstruction techniques, and its frequent clinical presentation with cacosmia indicate that postoperative osteitis is more than a nonspecific sinonasal complication. These observations warrant a focused discussion on the potential pathophysiological mechanisms, the role of surgical technique and reconstruction materials, and the clinical implications of sphenoid sinus osteitis in the postoperative period.

Endoscopic transsphenoidal surgery (ETS) has become the standard approach for the treatment of pituitary and adjacent sellar–parasellar lesions [4, 17, 34]. Despite its minimally invasive nature, postoperative sinonasal and skull base–related complications may significantly affect patient comfort, quality of life, and long-term surgical outcomes [7, 22]. In the existing literature, infectious complications following ETS have predominantly been discussed under the umbrella of sinusitis, mucosal inflammation, or nasal cavity–related disorders, whereas inflammatory involvement of the sphenoid sinus bony walls—namely osteitis—has rarely been addressed as a distinct pathological entity [1, 22]. To our knowledge, the present study represents the first large patient series systematically investigating the incidence, clinical manifestations, and surgery-related risk factors of sphenoid sinus osteitis following endoscopic transsphenoidal surgery.

The development of osteitis is a multifactorial process, with periosteal injury secondary to surgical trauma, local bone ischemia, and inflammatory responses to reconstruction materials constituting the main contributing factors [1, 11, 20]. In the chronic rhinosinusitis literature, osteitis has been defined as an inflammatory process involving the bony framework, characterized by sclerosis, cortical thickening, and neo-osteogenesis [19, 20, 28]. The radiological osteitis criteria proposed by Lee and Kennedy provide an imaging-based representation of this inflammatory process [29].

In endoscopic skull base surgery, collagen-based dural substitutes, autologous fascia lata grafts, and vascularized nasoseptal flaps are widely used for sellar reconstruction to reduce the risk of cerebrospinal fluid (CSF) leakage; however, direct contact of these materials with the underlying bone surface may trigger a chronic, low-grade inflammatory response [3, 13, 24, 30, 34]. In particular, extensive contact of a vascularized nasoseptal flap with the sphenoid sinus floor may induce a bone remodeling process characterized by regional hyperemia, increased osteoblastic activity, and neo-osteogenesis [13, 31]. The exclusive occurrence of osteitis in patients who underwent sellar reconstruction in our cohort strongly supports the pivotal role of surgical reconstruction in the pathogenesis of postoperative sphenoid sinus osteitis.

In neurosurgical practice, the terms “osteitis” and “osteomyelitis” are commonly used to describe infections of free bone flaps following craniotomy or complications related to cranioplasty [12, 14, 35]. In such settings, devascularized bone flaps, the presence of foreign materials, and high-grade infection typically predominate, and the condition is generally considered within the spectrum of osteomyelitis [14, 35]. In contrast, osteitis involving the sphenoid sinus floor following endoscopic transsphenoidal surgery represents a pathophysiologically distinct entity. The compact structure of the sphenoid bone, its limited bone marrow content, and the preservation of surrounding vascular connections—unlike intracranial free bone flaps—appear to favor a localized osteitis pattern rather than overt osteomyelitis [17, 25]. This distinction is clinically relevant both for accurate terminology and for appropriate management strategies.

Although positron emission tomography/computed tomography (PET/CT) was not routinely used in this study, increased FDG uptake supporting the diagnosis of osteitis was observed in a limited number of patients with diagnostic uncertainty. Previous reports have indicated that PET/CT may serve as a useful adjunct in differentiating chronic osteitis from osteomyelitis [18, 23].

Postoperative sinonasal complications reported after ETS primarily include sinusitis, nasal crusting, mucosal edema, and infection, while cacosmia has often been regarded as a secondary or nonspecific symptom [7, 10]. However, the presence of cacosmia in 75% of patients diagnosed with osteitis in our series suggests that inflammatory involvement of the sphenoid sinus bone may play a significant role in the development of this symptom. Chronic inflammation associated with osteitis may impair local mucosal healing, promote secretion stasis, and facilitate low-grade infection, thereby contributing to the perception of foul odor [2, 15, 21]. These findings indicate that, in patients presenting with persistent or late-onset cacosmia after endoscopic transsphenoidal surgery, radiological evaluation should extend beyond mucosal pathology to include assessment of the underlying bony structures.

From a management perspective, all patients with osteitis initially received a standardized medical regimen consisting of two courses of oral clarithromycin (each lasting 10 days) and saline nasal irrigation three times daily. Despite this initial treatment, surgical revision was ultimately required in 11 of 12 patients (91.7%), suggesting that sphenoid sinus osteitis may respond poorly to medical therapy alone. Only one patient achieved complete symptom resolution with medical treatment alone. These findings suggest that osteitis, once established, may require direct surgical intervention to adequately control the inflammatory process [5, 21].

Microbiological evaluation revealed a culture positivity rate of 33.3%, with Staphylococcus aureus being the most frequently isolated organism. The presence of pronounced radiological osteitis findings in culture-negative cases raises the possibility of sterile inflammation or biofilm-associated low-grade infection independent of overt microbial growth [9, 18]. The role of biofilm formation in the pathogenesis of chronic rhinosinusitis and osteitis has been previously demonstrated, and similar mechanisms may contribute to sphenoid sinus osteitis [6, 26]. Furthermore, osteitic processes originating from the sphenoid region have, albeit rarely, been reported to progress to serious intracranial complications [26].

Although the nasoseptal flap reached statistical significance (*p* = 0.045), this borderline finding should be interpreted with caution due to the limited number of cases, which precludes a definitive assertion of superiority over other reconstruction techniques. Nevertheless, osteitis occurred exclusively in patients who underwent sellar reconstruction, all of which involved the use of fibrin sealant, suggesting—consistent with existing experimental evidence—that contact between fibrin sealant and exposed bone may contribute to localized inflammatory or osteitic processes [8, 27]. Meticulous positioning of the vascularized nasoseptal flap, ensuring precise coverage of the sellar defect while avoiding unnecessary or excessive contact with adjacent exposed bony surfaces, may reduce persistent inflammatory stimulation and thereby limit reactive bone remodeling at the sphenoid sinus floor. These technical nuances suggest that while reconstruction is often essential, its execution must be tailored to balance effective closure with the preservation of underlying bony integrity.

Experimental studies suggest that fibrin sealants, while generally considered biocompatible, may be associated with localized inflammatory changes when applied to sinonasal tissues. In particular, prolonged contact between fibrin-based materials and exposed bone could potentially contribute to low-grade inflammatory responses, submucosal fibrosis, or reactive bone remodeling, supporting the possibility of a bone-centered process rather than a purely mucosal reaction [8, 27].

Taken together, the findings of the present study and the existing literature on endoscopic skull base surgery suggest that no single surgical maneuver can completely prevent the development of osteitis during endoscopic transsphenoidal surgery. These observations support the concept that sphenoid sinus osteitis is a reconstruction-driven, bone-centered inflammatory entity, triggered by persistent contact between reconstruction materials and exposed sphenoid bone. Nevertheless, attention to certain technique-related factors may potentially reduce the risk [4, 22, 34]. Avoiding unnecessary extensive bone exposure of the sphenoid sinus floor, preserving periosteal integrity whenever possible, and positioning the vascularized nasoseptal flap to cover only the required area without excessive bone contact appear to be particularly important [1, 20]. In addition, tailoring the sellar reconstruction strategy according to defect size and CSF leakage risk may help avoid unnecessary use of reconstruction materials [3, 16, 24, 30]. Although these measures may not entirely prevent osteitis, they may contribute to reducing the risk of chronic low-grade inflammation within the sphenoid sinus floor [1, 11, 17]. Prospective studies are required to validate these recommendations.

The main limitations of this study include its retrospective design and the relatively small number of osteitis cases. Although all procedures were performed within a standardized institutional framework, variability in surgical technique among surgeons cannot be entirely excluded and may have influenced the observed outcomes. However, this variability also reflects real-world clinical practice, thereby enhancing the generalizability of our results. Histopathological confirmation was not available, and advanced imaging modalities were used only in selected patients. Nevertheless, the large patient cohort and systematic radiological evaluation constitute notable strengths of the study. Future research should focus on prospective designs, histopathological and microbiological correlation, long-term assessment of sinonasal functional outcomes (such as NOSE scores and objective olfactometry), and comparative analyses of the biological effects of different reconstruction materials on bone tissue, which may further elucidate sphenoid sinus osteitis following endoscopic transsphenoidal surgery.

## Conclusion

Sphenoid sinus osteitis following endoscopic transsphenoidal surgery is an uncommon but clinically relevant complication. In this study, osteitis occurred exclusively in patients who underwent sellar reconstruction, suggesting a potential association between reconstruction techniques and the development of this pathology. Direct contact between reconstruction materials and the sphenoid sinus floor, together with a localized inflammatory response, may represent a key underlying mechanism.

Cacosmia emerged as the most frequent and clinically distinctive symptom in affected patients. These findings indicate that persistent or delayed-onset olfactory disturbances after endoscopic transsphenoidal surgery should prompt evaluation of the underlying bony structures in addition to routine assessment of sinonasal mucosa. Although surgical revision was required in the majority of patients, treatment outcomes were favorable, with most patients achieving complete symptom resolution at 12-month follow-up.

Overall, this study identifies sphenoid sinus osteitis as a distinct, bone-involving postoperative entity rather than a conventional rhinonasal complication and highlights the need for heightened awareness in clinical practice. By systematically characterizing this complication in a large surgical cohort, the present work addresses an important gap in the literature and provides a framework for future studies aimed at refining preventive strategies and optimizing postoperative management. Recognizing sphenoid sinus osteitis as a distinct postoperative entity may improve diagnostic accuracy and guide more targeted management of postoperative rhinonasal symptoms.

## Data Availability

No datasets were generated or analysed during the current study.
